# Immune Activation at Sites of HIV/TB Co-Infection Contributes to the Pathogenesis of HIV-1 Disease

**DOI:** 10.1371/journal.pone.0166954

**Published:** 2016-11-21

**Authors:** Qinglai Meng, Ismail Sayin, David H. Canaday, Harriet Mayanja-Kizza, Joy Baseke, Zahra Toossi

**Affiliations:** 1 Division of Infectious Disease, Case Western Reserve University, Cleveland, Ohio, United States of America; 2 Veterans Affairs Medical Center, Cleveland, Ohio, United States of America; 3 Department of Medicine, Makerere University, Kampala, Uganda; 4 Joint Clinical Research Center, Kampala, Uganda; University of Texas Rio Grande Valley, UNITED STATES

## Abstract

Systemic immune activation is critical to the pathogenesis of HIV-1 disease, and is accentuated in HIV/TB co-infected patients. The contribution of immune activation at sites of HIV/TB co-infection to viral activity, CD4 T cell count, and productive HIV-1 infection remain unclear. In this study, we measured markers of immune activation both in pleural fluid and plasma, and in T cells in pleural fluid mononuclear cell (PFMC) and peripheral blood mononuclear cell (PBMC) in HIV/TB co-infected subjects. The relationship between soluble and T cell activation markers with viral load in pleural fluid and blood CD4 T cell count were assessed. The T cell phenotype and activation status of HIV-1 p24 + T cells in PFMC and PBMC from HIV/TB patients were determined. We found that T cell and macrophage-specific and non-specific soluble markers of immune activation, sCD27, sCD163, IL1Ra, and sCD14, were higher in pleural fluid as compared to plasma from HIV/TB co-infected subjects, and higher as compared to pleural fluid from TB mono-infected subjects. Intestinal fatty acid-binding protein, a marker of intestinal tract damage, in plasma from HIV/TB co-infected patients was not different than that in HIV+ subjects. Expression of HLADR and CD38 double positive (HLADR/CD38) on CD4 T cells, and CD69+ on CD8 T cells correlated with pleural fluid viral load, and inversely with blood CD4 T cell count. Higher expression of HLADR/CD38 and CCR5 on CD4 T cells, and HLADR/CD38 and CD69 on CD8 T cells in PFMC were limited to effector memory populations. HIV-1 p24+ CD8 negative (includes CD4 + and double negative T cells) effector memory T cells in PFMC had higher expression of HLADR/CD38, Ki67, and CCR5 compared to HIV-1 p24- CD8 negative PFMC. Cumulatively, these data indicate that sites of HIV/TB co-infection are the source of intense immune activation.

## Introduction

A central role for systemic immune activation in the pathogenesis of HIV-1 disease has long been recognized. Significant associations between T cell activation and viral load, CD4 T cell loss, and mortality, have been demonstrated [1–3]. Circulating markers of systemic immune activation predict mortality in HIV-1 disease both in anti-retroviral therapy (ART) treated [4] and untreated [5] subjects. Microbial translocation originating from damaged gastrointestinal lymphoid tissue (GALT) underlies systemic immune activation during HIV-1 disease in part [6, 7]. However, the basis of immune activation during co-infections of HIV-1 disease, and its contribution to promotion of HIV-1 infection is less well understood.

Tuberculosis (TB) is the most common opportunistic infection during HIV-1 disease worldwide [8]. Development of TB accelerates progression of HIV-1 disease and promotion of mortality [9]. Higher HIV-1 viral loads have been consistently found at sites of active HIV/TB co-infection compared to peripheral blood [10, 11]. Studies on HIV-1 infected subjects with pleural TB indicate that viral load in pleural fluid correlates with HIV-1 mRNA in pleural fluid mononuclear cells (PFMC) [12], and with the frequency of HIV-1 p24 positive T cells among PFMC [13]. Higher HIV-1 genetic heterogeneity in pleural fluid as compared to that in the plasma of HIV/TB co-infected patients [11] further corroborates pleural sites as the main site of HIV-1 replication at the time of diagnosis of TB. However, the contribution of immune activation at pleural sites of HIV/TB co-infection to pathogenesis of HIV-1 disease has not been studied in depth.

Several soluble markers of systemic immune activation have been shown to correlate with the course of HIV-1 disease. Significantly higher levels of circulating soluble CD14 (sCD14), a non-specific marker of macrophage activation [14], were found in HIV/TB co-infected patients with pulmonary TB as compared to CD4-matched HIV-1 infected healthy subjects, that was irrespective of their CD4 T cell count [15]. In this latter study, only in HIV/TB co-infected patients with high CD4 T cell counts (over 350/μl), plasma sCD14 and the more macrophage-specific hemoglobin scavenger molecule, sCD163, decreased to levels detected in HIV-1 infected control subjects upon completion of TB treatment [15]. These data implicate that sites of active HIV/TB co-infection are dominant in contribution to systemic immune activation.

The contribution of microbial translocation from GALT to systemic immune activation in HIV-1 infected patients with opportunistic infections appears to differ according to the site of co-infection. For example, in HIV-1 infected patients with Visceral Leishmaniasis, heightened microbial translocation was associated with intense GALT-associated immune activation [16]. By contrast, in the study of HIV/TB co-infected patients with pulmonary TB, a dissociation of microbial translocation (measured as plasma LPS) and systemic immune activation was found [15], implicating other factors contributing to immune activation during HIV/TB co-infection.

Both activated and resting CD4 T cells express HIV-1 RNA in lymph node and peripheral blood from HIV-1 infected subjects, and not surprisingly the intensity of HIV-1 expression is much higher in activated as compared to resting cells [17, 18]. Among PFMC from HIV/TB co-infected subjects with pleural disease, CD4-CD8- double negative (DN) T cells derived from CD4 T cells are the major cells with active HIV-1 production [13]. However, the activation status of T cells with productive HIV-1 infection (i.e. CD4+ and DN T cells), or CD8 T cells at sites of HIV/TB co-infection have not been characterized.

In this study, we compared levels of soluble and T cell markers of immune activation between systemic and the pleural site of HIV/TB co-infection at the time of diagnosis and in the absence of anti-retroviral therapy (ART), and analyzed the relationship between soluble and T cell activation markers with pleural fluid viral load and peripheral blood CD4 T cell count. We then defined the T cell phenotype and activation status of HIV-1 p24^+^ T cells in PFMC from HIV/TB co-infected patients.

## Methods

### Subjects with pleural tuberculosis

Symptomatic patients with fever, cough, night sweats for at least 2 weeks that were hospitalized at Mulago Hospital (Kampala, Uganda) and had chest x-ray evidence of moderate to large pleural effusion were recruited. All subjects were HIV-1 tested and underwent thoracentesis and sputum examination for diagnosis of TB. Some subjects had pleural biopsy as described before also [19]. The study and the written informed consent were approved by the Institutional Review Boards at Makerere University (Kampala, Uganda) and Case Western Reserve University/University Hospitals (Cleveland, Ohio). All subjects diagnosed with TB received treatment for TB, and HIV-1 infected subjects were referred for ART to Mulago Joint AIDS Program Clinic. HIV-1 infected and un-infected patients who were culture positive for *M*. *tuberculosis* (MTB) in pleural biopsy specimens, pleural fluid or sputum were included in this study.

A group of Ugandan asymptomatic HIV-1 mono-infected (HIV+) and healthy (HS) subjects were recruited also. Clinical characteristics of subjects are provided in Table 1.

**Table 1:** Characteristics of study subjects.

Patient group	Number	Age	Gender	Blood CD4 count	Viral load(copy/ml)	
		Median(range)	(Male/Female)	Median(range)	plasma	pleural fluid
**HIV/TB co-infected**	**25**	**35(25–55)**	**17/8**	**190(32–650)**	**190,029**	**1,226,000**
					**(473–988,740)**	**(18,286–6,738,000)**
**HIV mono-infected**	**13**	**38(24–65)**	**4/9**	**293(174–463)**	**26,378**	**N/A**
					**(1,892–31,6574)**	
**TB mono-infected**	**11**	**28(23–49)**	**8/3**	**N/A**	**N/A**	**N/A**
**Healthy subjects**	**13**	**32(23–59)**	**6/7**	**N/A**	**N/A**	**N/A**

### Preparation of mononuclear cells

PFMC and PBMC were prepared from paired pleural fluid and blood at the time of clinical evaluation as before [10]. Cryopreserved PFMC and PBMC were prepared in Uganda and transported to CWRU along with frozen pleural fluid and plasma. Mononuclear cell samples were thawed in CTL anti-aggregate wash buffer (Cellular Technology Limited, Shaker Heights, OH) according to the manufacturer’s instructions.

### Soluble markers of Immune activation

Plasma and pleural fluid were examined by the following enzyme linked immune assays (ELISA) according to the instruction of the manufacturers. Soluble (s) CD14 and sCD163 (R&D systems, Minneapolis, MN), IL1-Ra and sCD27 (e-biosciences, San Diego, CA), and Intestinal Fatty acid Binding Protein (IFABP) (Hycult Biotech, Plymouth, PA).

### Flow cytometric analysis

PFMC or PBMC were stained with LIVE/DEAD yellow fixable dead cell stain kit (Life Technology, Eugene, OR) and with the following two monoclonal antibody staining panels: In Panel 1, cells were surface-stained by anti-CD3-PerCP, CD4-PE-Cy7, CD45RO-FITC, CD69-APC, CD38-AF700 (BD Bioscience, San Jose, CA), CD8-PE-TR (Invitrogen, Frederick, MD), CCR7-APC-Cy7 and HLADR-Pacific Blue (BioLegend, San Diego, CA). Cells were then fixed, permeabilized, and washed with Transcription Factor Buffer Set (BD Pharmingen) according to the manufacturer and stained with anti-HIV-1 p24-PE (KC57) (Beckman Coulter, Indianapolis IN). In Panel 2, monoclonal antibodies included: anti CD25-APC (BioLegend), CCR5-V450 and intracellular staining for Ki67-BV711 (both from BD Bioscience) in addition to anti-CD3, CD4, CD8, CCR7, CD45RO, and HIV-1 p24 as in panel 1. Gates were set using Fluorescent-Minus-One controls for each sample. T cells were identified as naïve (CD45RO-CCR7+), central memory (Tcm) (CD45RO+CCR7+), effector memory (Tem) (CD45RO+CCR7-), and terminally differentiated effector memory (TemRA) (CD45RO-CCR7-). Stained samples were analyzed by a LSRII cytometer (BD). Data were analyzed using FlowJo (Tree Star, Ashland, OR).

### Statistical analysis

Statistical analyses were performed with Prism (GraphPad Software Inc, San Diego, CA). All data were compared by the non-parametric Wilcoxon rank test or Mann Whitney U t test, and a P value less than 0.05 was considered significant. Spearman’s correlation coefficient was used to determine the correlation between variables.

## Results

### Soluble markers of immune activation at sites of HIV/TB co-infection

**Fig 1:**
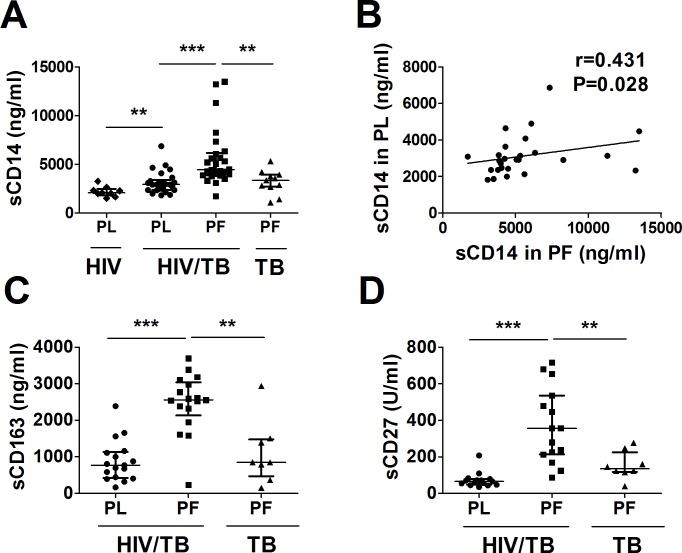
Soluble Markers of immune activation at sites of HIV/TB co-infection. Plasma (PL) and Pleural fluid (PF) from HIV/TB co-infected subjects were assessed for soluble activation markers and compared to PL from HIV-1 infected and PF from TB mono-infected subjects. (**A**) sCD14 levels in PL and PF, (**B**) sCD14 correlation between PF and PL, (**C**) sCD163 and (**D**) sCD27 levels in PL and PF. **, p **<** 0.01 and ***, p < 0.001

First, we assessed soluble markers of immune activation in pleural fluid and plasma from HIV/TB co-infected patients. Pleural fluid from TB mono-infected patients was included in each analysis (Fig 1A, 1C and 1D). Plasma from HIV + asymptomatic subjects were included in analysis of sCD14 also (Fig 1A).

Pleural fluid sCD14 was higher than autologous plasma in HIV/TB patients (Fig 1A). Pleural fluid sCD14 was significantly higher in HIV/TB co-infected as compared to TB mono-infected patients also. In addition, plasma sCD14 from HIV/TB co-infected was higher than healthy HIV-1 + subjects. Therefore, immune activation is specifically augmented at sites of HIV/TB co-infection. Further, similar to subjects with pulmonary HIV/TB co-infection [15], systemic immune activation was significantly higher in plasma from patients with pleural HIV/TB co-infection as compared to HIV-1 mono-infected subjects. Importantly, sCD14 in pleural fluid correlated significantly with sCD14 in plasma (Fig 1B), underscoring the contribution of immune activation at sites of HIV/TB co-infection to systemic immune activation.

Next, we assessed IL1 receptor antagonist (IL1Ra) in pleural fluid and plasma from a subgroup HIV/TB co-infected subjects (n = 9) to further confirm excessive immune activation in situ. IL1Ra is a cell type non-specific marker of immune activation, and is expressed by the same cells that express IL-1 [20] and in concert with it [21]. Levels of IL1Ra in pleural fluid were 2.39 fold higher than in plasma (S1A Fig), and pleural fluid IL1Ra correlated with that in plasma (S1B Fig). In addition, IL1Ra levels in pleural fluid correlated with viral load both in the pleural fluid (S1C Fig) and plasma (S1D Fig). However, pleural fluid IL1Ra levels did not correlate with sCD14.

Recently, assays that identify the mononuclear cell type origin of immune activation more specifically, sCD163 (for monocyte/macrophages) and sCD27 (for T cells) have been described [22–24]. Here, we analyzed plasma and pleural fluid from patients with HIV/TB co-infection for both sCD163 (n = 17) and sCD27 (n = 15). Pleural fluid from HIV-1 un-infected TB patients (n = 8) was assessed also. As shown in Fig 1C and 1D, pleural fluid from HIV/TB co-infected patients contained significantly higher sCD163 (2.81 fold, p<0.001) and sCD27 (5.1 fold, p<0.001) as compared to autologous plasma. Also, both molecules were significantly higher in pleural fluid from HIV/TB co-infected as compared to that from TB mono-infected patients. Pleural fluid sCD163 correlated with plasma sCD163 (r = 0.55, p<0.05). However, pleural fluid sCD27 did not correlate with its plasma levels. Also, pleural fluid sCD163 did not correlate with sCD14.

Cumulatively, these data indicate that soluble markers of immune activation, including both T cell and macrophage-specific and non-specific molecules, are higher in pleural compartment as compared to systemically in HIV/TB co-infected subjects. Also, in situ immune activation is significantly higher in HIV/TB co-infection as compared to TB mono-infection.

### Lack of evidence for increased microbial translocation during HIV/TB co-infection

**Fig 2:**
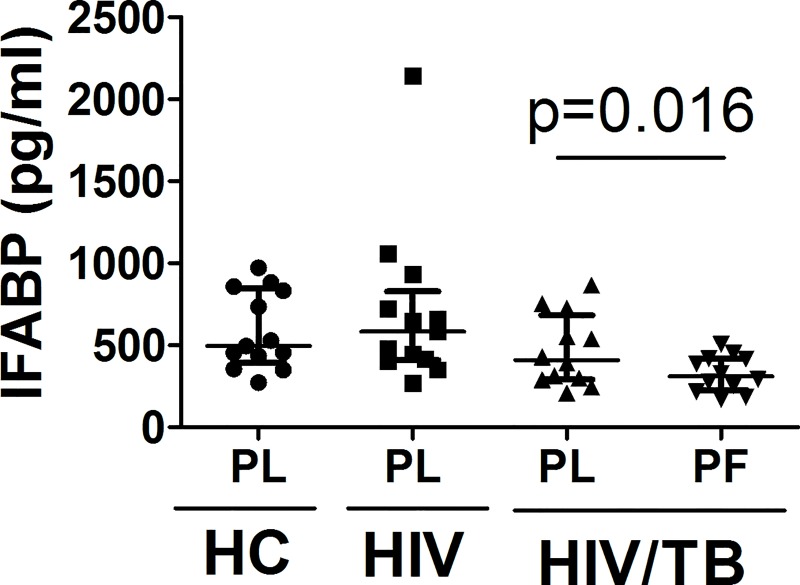
IFABP in HIV/TB subjects with pleural TB. IFABP levels were measured in pleural fluid (PF) and plasma (PL) from HIV/TB co-infected patients, and in PL from CD4-matched HIV-1 infected (HIV) and HIV-1 un-infected healthy (HC) subjects.

Intestinal fatty acid binding protein (IFABP), a recently described marker of inflammation and damage to GALT [25], has been found to correlate significantly with microbial translocation and systemic immune activation during HIV-1 disease [26]. Here, we measured IFABP in plasma and pleural fluid from a subgroup (n = 12) of HIV/TB co-infected subjects with pleural disease, and compared it to plasma from a group of CD4 T cell matched healthy HIV-1 infected subjects with no evidence of TB (n = 13) (Fig 2). Plasma from a group of healthy HIV-1 sero-negative subjects (n = 13) age-matched to HIV-1 infected subjects were included also. IFABP levels in plasma from HIV/TB co-infected patients were not significantly different from that of healthy HIV-1 infected subjects without TB. However, as expected plasma IFABP levels were higher than that in autologous pleural fluid (p<0.02) (Fig 2), which likely reflects diffusion of IFABP from plasma to the pleural site of HIV/TB co-infection. Surprisingly, in this cohort from Africa, plasma IFABP levels in HIV-1 infected healthy subjects were not significantly higher when compared to healthy age-matched HIV-1 un-infected subjects.

### Expression of activation markers on PFMC T cells from HIV/TB patients

**Fig 3:**
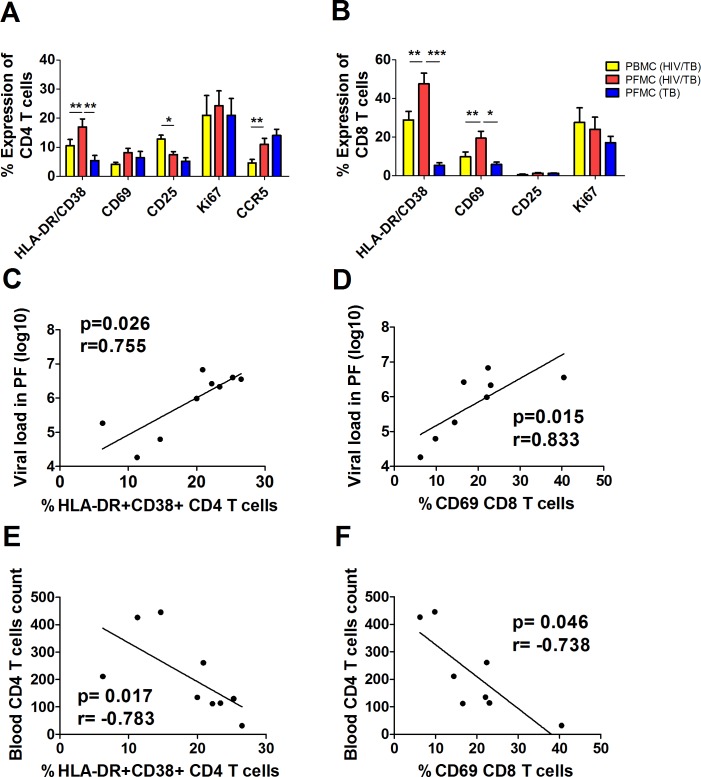
Expression of markers of immune activation and proliferation on CD4 and CD8 T cells at sites of HIV/TB, and association with HIV viral load in pleural fluid and CD4 T cell counts. Markers of immune activation and proliferation were assessed on PFMC T cells as compared to PBMC from HIV/TB co-infected subjects, and between PFMC from HIV/TB and TB mono-infected subjects. Analysis of CD4+ (**A**) and CD8+ (**B**)T cells are shown. Association of HLADR/CD38 on PFMC CD4 (**C and E**) and CD69 on PFMC CD8 (**D and F**) T cells with HIV viral load in pleural fluid (PF) **(C and D)** and blood CD4 T cell count (**E and F**). *, p< 0.05; **, p< 0.01 and ***, p**<** 0.001.

Next, we compared the activation and proliferation profile of CD4 and CD8 T cells between PFMC and PBMC from HIV/TB co-infected subjects. PFMC from TB mono-infected patients were assessed in parallel also. S2 Fig is an algorithm for analysis of activation and proliferation of PFMC and PBMC T cells. As shown in Fig 3A, PFMC CD4 T cells from HIV/TB subjects expressed significantly higher HLADR and CD38 double positive (HLADR/CD38), and CCR5 as compared to PBMC. Further, expression of CD69, and HLADR/CD38 were significantly higher on CD8 PFMC T cells than PBMC (Fig 3B). Expression of HLADR/CD38 was significantly higher in both CD4 and CD8 PFMC T cells from HIV/TB co-infected subjects as compared to TB mono-infected subjects. Of note, Ki67+ CD4 or CD8 cells were equally high in both PFMC and PBMC from HIV/TB patients, and similar between PFMC from HIV/TB and TB mono-infected subjects.

Next, the correlation of activation markers on CD4 and CD8 T cells in PFMC with pleural fluid HIV-1 viral load and blood CD4 T cell counts was assessed. Median HIV-1 viral load was almost 10 fold higher in pleural fluid than in plasma of patients with HIV/TB (Table 1) (p<0.0001). HIV viral load in pleural fluid correlated significantly with expression of HLADR/CD38 on PFMC CD4 T cells (Fig 3C) and expression of CD69 on PFMC CD8 T cells (Fig 3D). In addition, pleural fluid viral load correlated with expression of Ki67 in PFMC CD4 T cells (r = 0.7, p<0.05), and with expression of HLADR/CD38 on CD8 T cells (r = 0.762, p<0.05) (not shown).

To understand the relationship between T cell activation at pleural sites of active HIV/TB co-infection with the hallmark of HIV-1 disease, i.e. CD4 lymphocytopenia, we analyzed the relationship between activation markers on CD4 and CD8 T cell in PFMC with blood CD4 T cell counts. Co-expression of HLADR/CD38 on CD4 T cells and expression of CD69 on PFMC CD8 T cell were inversely associated with CD4 T cell count (Fig 3E and 3F).

### Differentiation and immune activation of Tem among PFMC in HIV/TB co-infection

**Fig 4:**
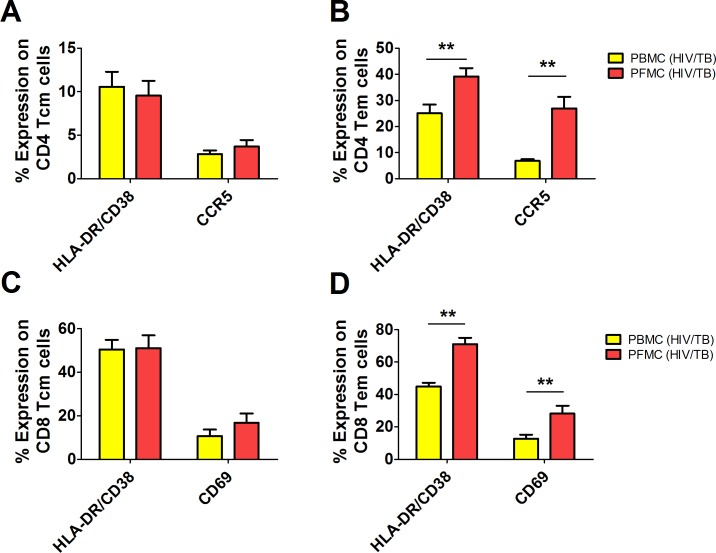
Expression of markers of immune activation on central and effector memory CD4 and CD8 T cells in PFMC. Expression of activation markers (HLADR/CD38, CCR5, CD69) was assessed on central memory (Tcm) (**A** and **C**) and effector memory (Tem) (**B** and **D**) for CD4 (**A** and **B**) and CD8 (**C** and **D**) in PBMC and PFMC T cells from HIV/TB co-infected subjects. **, p **<** 0.01 and ***, p < 0.001

Next, we analyzed expression of activation markers on CD45RO+ PFMC and PBMC T cells based on co-expression of CCR7 (T cm) or lack of it (Tem) in HIV/TB co-infected subjects. S3 Fig is an algorithm for analysis of activation markers on CD45RO+ CD4 and CD8 T cells. Results for CD4 T cells (Fig 4A and 4B) and CD8 T cells (Fig 4C and 4D) are shown. Differences in expression of activation markers, HLADR/CD38 and CCR5, between PBMC and PFMC CD4 T cells were limited to Tem alone. Similarly, HLADR/CD38 and CD69 on CD8 Tem, but not Tcm, were higher in PFMC compared to PBMC.

In addition, frequency of Tem CD4 cells in PFMC inversely correlated with absolute CD4 T cell count in the blood (p<0.02, r = -0.783) (data not shown).

### Differentiation and activation characteristics of HIV-1-infected T cells in PFMC

**Fig 5:**
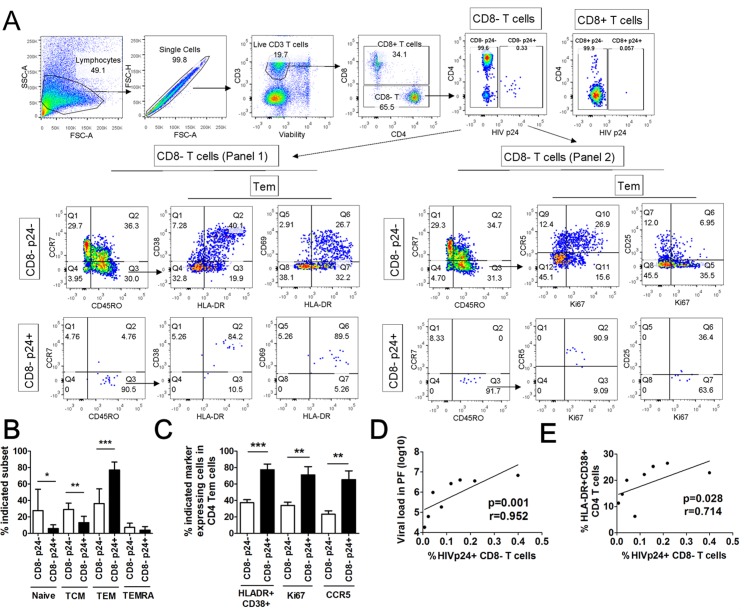
Activation and differentiation state of PFMC T cells with productive HIV-1infection. T cells were gated by expression of intracellular HIV-1 p24 and differentiation and activation markers (CD45RO, CCR7, HLADR, CD38), proliferation marker (Ki67) and CCR5. **(A)** A representative analysis of PFMC p24+ (lower panel) and p24- (upper panel) CD8- T cells is shown. **(B):** Comparative analysis of frequency of naïve, central memory (TCM), effector memory (TEM) and terminally differentiated (TEMRA) HIV-1 p24 positive and negative CD8- T cells. **(C)**: Analysis of HLADR/CD38, Ki67 and CCR5 on HIV-1 p24 positive (black bar) and negative CD8- T cells (white bar) (n = 8) are shown. Association of HIV-1 p24 +CD8—T cells with viral load in pleural fluid **(D**) and HLADR+/CD38+ dual positive CD4 T cells **(E)**. *, p<0.05, **, p<0.01 and ***, p<0.001.

Finally, we compared intracellular HIV-1 p24 expression in T cell subsets in PBMC and PFMC from HIV/TB co-infected patients, and assessed cell differentiation and activation status of HIV-1 infected T cells. HIV-1 p24+ T cells were rarely detectable in PBMC (<0.001%), while significantly expanded in PFMC. Fig 5A is an algorithm for analysis of HIV-1 infected PFMC T cells using two staining panels. As noted, CD8^+^ T cells were devoid of HIV-1 infection. HIV-1 p24 expressing PFMC were gated as CD8 negative T cells to include both DN and CD4^+^ T cells as before [13]. Among HIV-1 p24+ CD8 negative PFMC T cells, Tem were significantly higher in frequency than HIV-1 p24—Tem (Fig 5B). These data suggest an increased differentiation of HIV p24+ compared to HIV p24- PFMC T cells towards Tem phenotype. Expression of HLADR/CD38, Ki67 and CCR5 on HIV-1 p24+ CD8 negative Tem cells were significantly higher than HIV-1 p24- CD8 negative Tem cells (Fig 5C). Frequency of HIV-1 p24 expressing CD8 negative PFMC T cell was associated with pleural fluid viral load (Fig 5D), and co-expression of HLADR/CD38 on CD4 Tcells (Fig 5E). Therefore, at pleural sites of HIV/TB co-infection, HIV-1 is preferentially expressed by effector memory CD8 negative T cell subset, that express a high activation profile.

## Discussion

A major determinant of HIV-1 disease progression is systemic immune activation, which is the product of a complex relationship between viral replication, immune profile of mononuclear cells, and GALT associated microbial translocation and/or HIV-1 co-infections. Currently, the “set point” of T cell immune activation is recognized to be best predictive of mortality in HIV-1 disease [1]. We found that in HIV/TB co-infected patients immune activation was localized to sites of co-infection and contributed significantly to systemic immune activation. Higher soluble and cellular markers of immune activation at sites of pleural HIV/TB co-infection as compared to TB alone, underscore the interaction of HIV-1 and MTB. Promotion of immune activation in PFMC was limited to Tem, and correlated with both in situ viral load and blood CD4 T cell counts. Highly activated CD8 negative (comprised of DN and CD4+ T cells) Tem were found to be HIV-1 p24+ in PFMC. We have recently shown that HIV-1 infected DN T cells among PFMC are derived from infected CD4+ T cells, under the influence of HIV-1 accessory proteins, Nef and Vpu [13].

All soluble markers of immune activation, including the more macrophage specific, sCD163, and the T cell specific, sCD27, were higher in pleural fluid as compared to blood in HIV/TB co-infected subjects (Fig 1). Interestingly sCD163, IL1Ra and sCD14 in pleural fluid significantly correlated with their respective levels in autologous plasma, underscoring the contribution of pleural HIV/TB compartment to systemic immune activation. Levels of IL1Ra in pleural fluid correlated with both systemic and pleural fluid viral load. Further, immune activation of both CD4 and CD8 T cells among PFMC of HIV/TB co-infected subjects were found to correlate with viral load in situ and to peripheral CD4 lymphocytopenia. Collectively, these data underscore the contribution of sites of co-infection to systemic immune activation, and thereby to viral immunopathogenesis during HIV/TB disease.

Here, we found that plasma IFABP levels, which reflect damage to GALT, to be no different in HIV/TB co-infected subjects than in HIV-1 infected subjects alone. Together with the significantly higher soluble markers of inflammation in situ, these data underscore pleural sites of HIV/TB co-infection as the main anatomical compartment, and negate a dominant role for GALT associated microbial translocation, in immune activation in HIV/TB co-infection. Lack of a role for microbial translocation in systemic immune activation has been recently shown in primary HIV-1 infection [27], and may be attributable to activation of “innate immunity” [28] at primary sites of viral replication i.e. the lymph nodes. Additionally, plasma IFABP levels were not significantly higher in healthy HIV + subjects than in healthy HIV-1 un-infected subjects. This latter finding from African HIV-1 un-infected healthy subjects is different from that reported from European healthy subjects [27], however, considering the prevalence of gastro-intestinal bacterial and parasitic infections in Africa, higher levels of plasma IFABP may not be expected. In fact, a study from Ethiopia on healthy HIV un-infected subjects has identified heightened markers of systemic immune activation [29]. Whether prior damage to the GALT carries a higher risk for HIV-1 disease progression subsequent to HIV-1 infection is currently unknown. However, at least in un-infected Pigtail Macaques, pre-existing damage to GALT was associated with rapid progression to AIDS upon SIV infection[30]. These latter findings are of concern regarding rapid HIV-1 progression in recently infected populations in the developing world and need to be addressed in further studies.

Interestingly, only activated PFMC Tem (and not Tcm) i.e. (HLADR/CD38 + and CCR5+) CD4 and (HLADR/CD38 + and CD69+) CD8 T cells were higher when compared to autologous PBMC (Fig 4). Of note, activated CD4 and CD8 PFMC were higher in HIV/TB co-infected as compared to TB mono-infected subjects also (Fig 3A and 3B). HIV-1 p24+ CD8 negative Tem, that included DN and CD4 T cells, were characterized by higher expression of HLADR/CD38, Ki67, and CCR5 (Fig 5), all of which are markers of successful HIV-1 infection of CD4 T cells. Thus, productive viral infection is associated with promotion of both memory T cell differentiation and immune activation at sites of HIV/TB co-infection.

Sites of HIV/TB co-infection are characterized by excessive bio active TGF-β and low IL-17) [12], and abundant secreted and released components of HIV-1 and MTB. This cytokine and molecular profile likely underlies excessive local immune activation and promotion of viral pathogenesis during HIV/TB in situ. Therapies to counteract immune activation coupled with ART and anti-TB antibiotics are needed to appropriately manage HIV/TB co-infection.

## Supporting Information

S1 Fig IL1Ra in pleural fluid (PF) and plasma from HIV/TB co-infected subjects. PF and PL HIV/TB co-infected subjects (n = 9) were assessed for IL1Ra. **(A)** Concentration of IL1Ra in PF and PL, **(B)** Correlation between IL1Ra in PF and PL. Association of PF IL1Ra with HIV-1 viral load in PF **(C)** and PL **(D)** *, p< 0.05.
(TIF)

S2 Fig Gating strategy for analysis of expression of activation, proliferation markers and CCR5 by flow cytometry. A representative analysis of expression of activation (HLA-DR, CD38, CD69 and CD25) and proliferation (Ki67) markers and the co-receptor CCR5 on PFMC CD4 and CD8 T cells from a TB mono-infected subject.
(TIF)

S3 Fig Gating strategy for analysis of expression of activation, proliferation markers and CCR5 on Naïve, Tcm, Tem and Temra T cell subsets in PFMC and autologous PBMC from HIV/TB co-infected subjects. A representative analysis of expression of activation (HLA-DR, CD38, CD69 and CD25) and proliferation (Ki67) markers, and CCR5 on Naïve (CD45RO-CCR7+), Tcm (CD45RO+CCR7+), Tem (CD45RO+CCR7-) and Temra (CD45RO-CCR7-) subsets of CD4 and CD8 PFMC T cells from one HIV/TB co-infected subject.
(TIF)
