# Supplementary figures and images for: Immune Activation at Sites of HIV/TB Co-Infection Contributes to the Pathogenesis of HIV-1 Disease
Source: PLoS One. 2016 Nov 21;11(11):e0166954. doi: 10.1371/journal.pone.0166954 (PMC5117743; doi:10.1371/journal.pone.0166954)

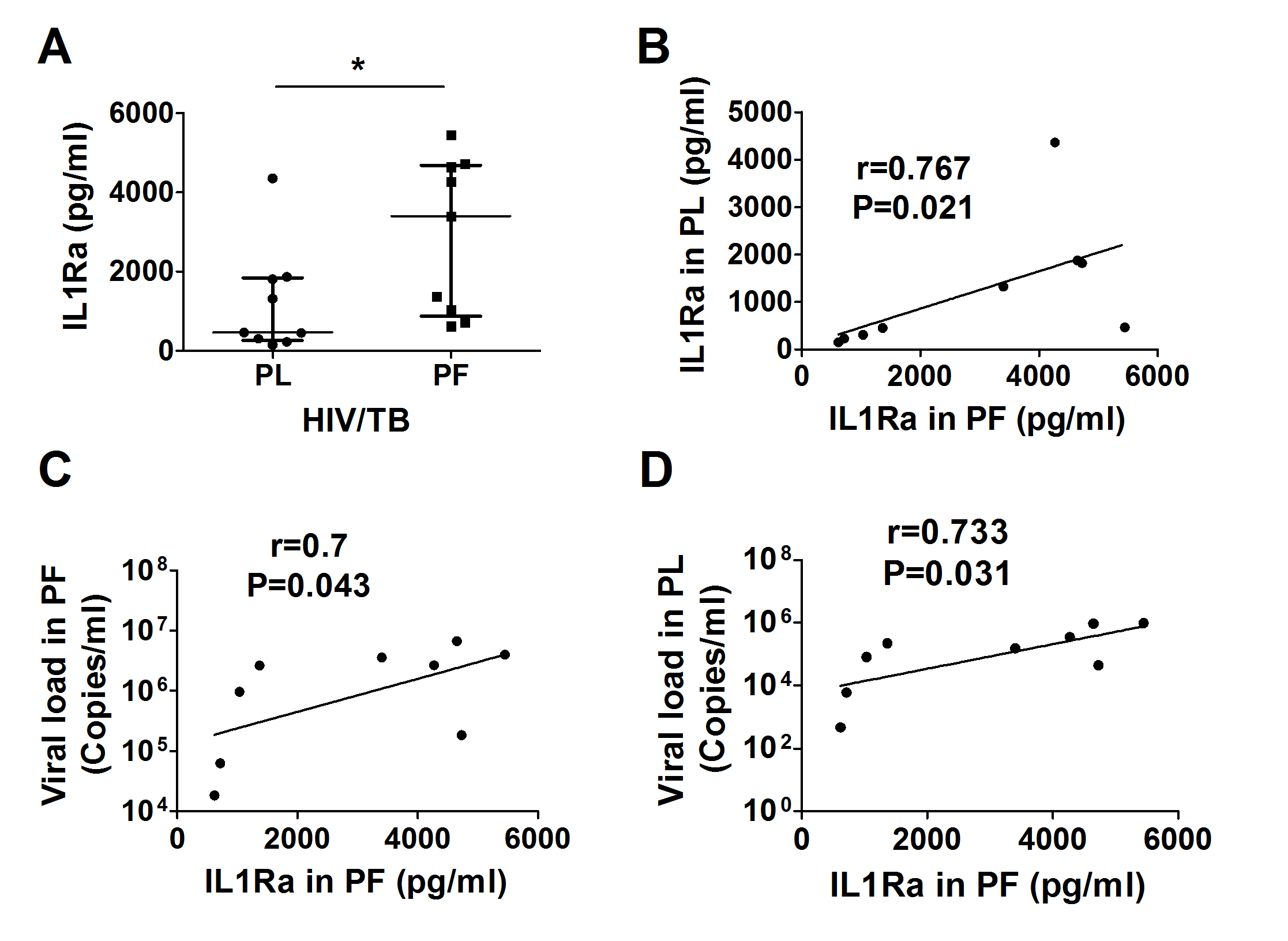

Supplement: S1 Fig — PF and PL HIV/TB co-infected subjects (n = 9) were assessed for IL1Ra. (A) Concentration of IL1Ra in PF and PL, (B) Correlation between IL1Ra in PF and PL. Association of PF IL1Ra with HIV-1 viral load in PF (C) and PL (D) *, p< 0.05. (TIF) [file pone.0166954.s001.tif]

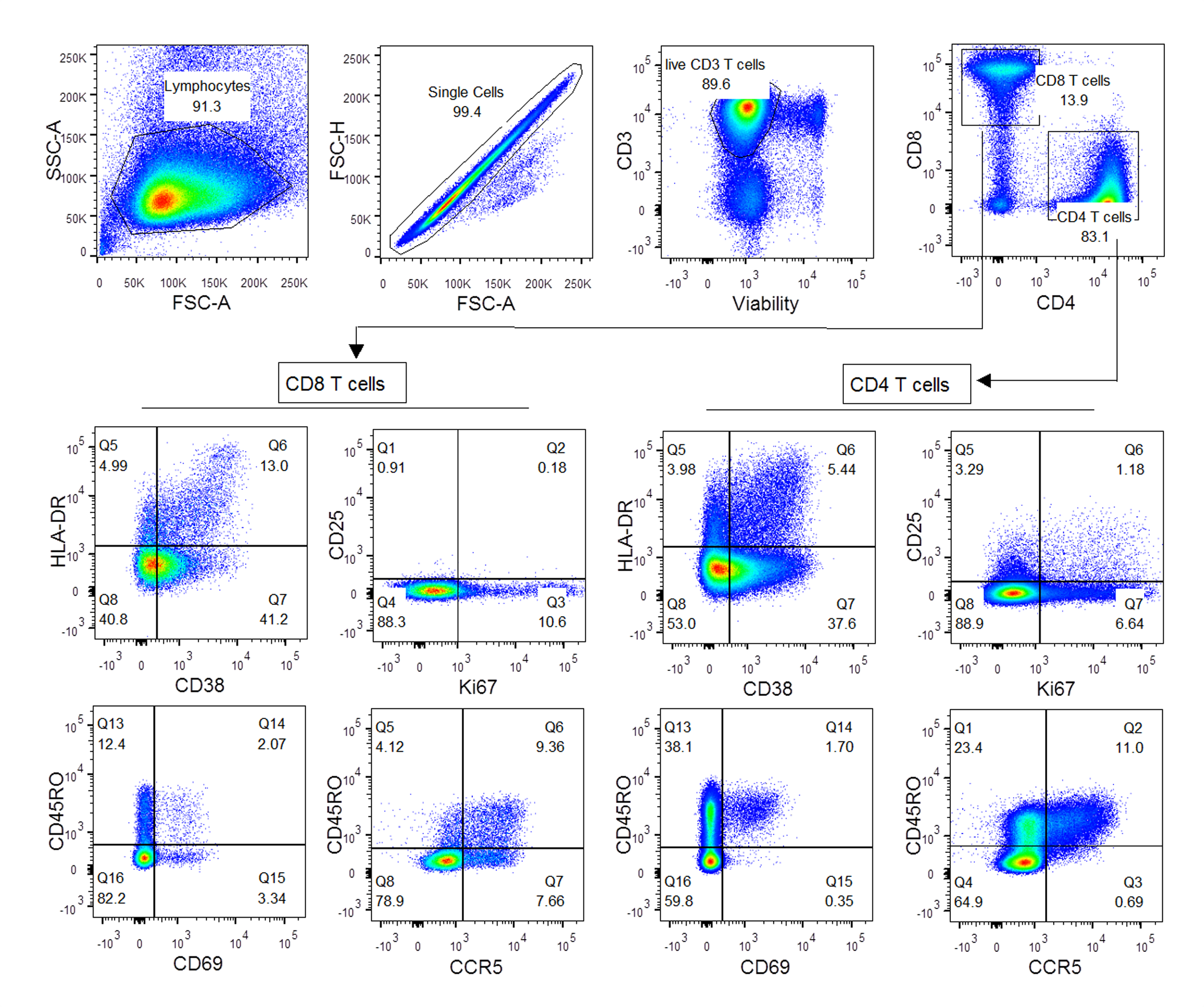

Supplement: S2 Fig — A representative analysis of expression of activation (HLA-DR, CD38, CD69 and CD25) and proliferation (Ki67) markers and the co-receptor CCR5 on PFMC CD4 and CD8 T cells from a TB mono-infected subject. (TIF) [file pone.0166954.s002.tif]

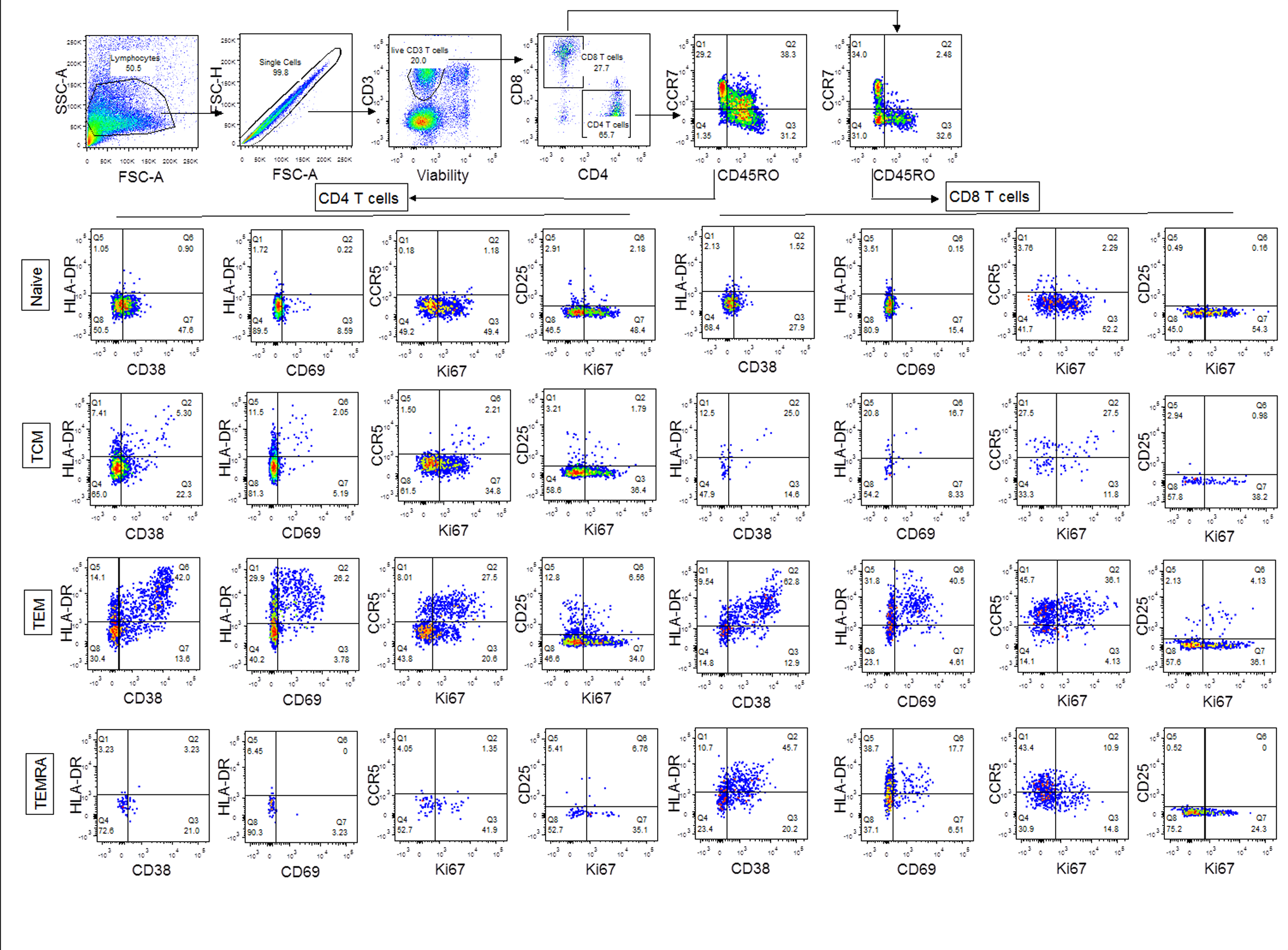

Supplement: S3 Fig — A representative analysis of expression of activation (HLA-DR, CD38, CD69 and CD25) and proliferation (Ki67) markers, and CCR5 on Naïve (CD45RO-CCR7+), Tcm (CD45RO+CCR7+), Tem (CD45RO+CCR7-) and Temra (CD45RO-CCR7-) subsets of CD4 and CD8 PFMC T cells from one HIV/TB co-infected subject. (TIF) [file pone.0166954.s003.tif]
